# Adaptation of an mHealth Solution for the Nutritional Management of Diabetes in a Low- and Middle-Income Country: Pre-Post Mixed Methods Pilot Study

**DOI:** 10.2196/58029

**Published:** 2025-09-25

**Authors:** Cecilia Anza-Ramirez, Karen Bonilla-Aguilar, David Beran, Jean-Luc Mando, Lorena Saavedra-Garcia, Monica Julissa Angulo-Barranca, Marco Alexi Taboada Garcia, Leonardo Albitres-Flores, Alejandro Loayza, Jessica Hanae Zafra-Tanaka, Olivia Heller, María Lazo-Porras, Montserrat Castellsague Pique Perolini

**Affiliations:** 1 CRONICAS Center of Excellence in Chronic Diseases, Universidad Peruana Cayetano Heredia, Av. Armendáriz 445, Miraflores, Lima, 18, Peru, 51 1 4448685; 2 Division of Tropical and Humanitarian Medicine, University of Geneva and Geneva University Hospitals, Geneva, Switzerland; 3 KmKoncept Sàrl, Veyrier, Switzerland; 4 Pediatric Diabetes Reference Center, Nuna Integral Pediatrics Clinic, Lima, Peru; 5 Nutrition, Faculty of Science and Engineering, Universidad Peruana Cayetano Heredia, Lima, Peru; 6 EsSalud, Lima, Peru; 7 Nutrition and Dietetics, Universidad Peruana de Ciencias Aplicadas, Lima, Peru; 8 Division of Tropical and Humanitarian Medicine, Geneva University Hospitals, Geneva, Switzerland

**Keywords:** diabetes mellitus type 1, user-centered design, counting carbohydrates, eHealth, mobile applications, low-income countries, middle-income countries, diabetes, insulin, quality of life

## Abstract

**Background:**

Carbohydrate counting (CC) is vital for individuals living with type 1 diabetes mellitus (T1DM); yet, formal training is often lacking in many contexts. To bridge this gap, the parents of a person living with diabetes and a team at the Geneva University Hospital (HUG) developed WebDia, a free-access app that helps patients with T1DM assess meal carbohydrates and make informed decisions regarding insulin dosage. In the context of Peru, where dietary patterns and meal compositions may differ, customizing WebDia to suit the components of the Peruvian diet becomes particularly relevant.

**Objective:**

This study aimed to customize WebDia according to the composition of the Peruvian diet to facilitate CC, and to provide training to health care workers (HCWs), children and adolescents living with T1DM, and their caregivers in the proficient use of WebDia-Mundi (new version of the Swiss app WebDia adapted to other geographic contexts).

**Methods:**

A dietitian compiled a database of Peruvian foods and their carbohydrate content. This was reviewed by a Swiss nurse specialized in diabetes, a Peruvian pediatric endocrinologist, and 2 researchers. Validation was conducted with a small group of children and adolescents living with T1DM and their caregivers. Subsequently, a 3-day workshop was held in 3 Peruvian regions for HCW and children and adolescents living with T1DM. The first 2 days were a training course for HCW to gain knowledge in T1DM and learn CC skills. This was followed by a 1-day workshop involving HCW, children and adolescents living with T1DM, and their caregivers. At the end of the workshop and 3 months later, an evaluation was performed to assess the app’s usability, glycated hemoglobin, quality of life, and knowledge perception for children and adolescents living with T1DM and their caregivers. Furthermore, changes in knowledge among HCWs and overall workshop satisfaction were measured.

**Results:**

WebDia-Mundi was customized for the Peruvian context in 2022-2023. The training was attended by 25 HCWs, 25 children and adolescents living with T1DM, and 31 caregivers. Following the training, HCWs exhibited a significant 3.5-point increase in their knowledge of T1DM, while achieving positive results regarding the usability of WebDia-Mundi. Children and adolescents living with T1DM and their caregivers also reported a favorable perception of the ease of use and functionality of WebDia-Mundi, which enhanced their CC skills.

**Conclusions:**

This study underscores the importance of collaboration among multidisciplinary teams and the involvement of individuals with T1DM. Adapting mobile health solutions to new contexts and sharing experiences can help standardize this process.

## Introduction

Diabetes represents a global health challenge, directly contributing to 1.5 million deaths annually, with a significant proportion of these deaths occurring in low- and middle-income countries (LMICs) [1]. While type 2 diabetes mellitus constitutes most diabetes cases, there are still approximately 9 million individuals grappling with type 1 diabetes mellitus (T1DM) [2]. Nonetheless, the persistent barrier of the cost of insulin and the supplies needed to maintain treatment for T1DM has a direct impact on individuals’ daily lives [3].

A notable barrier within the health care system revolves around the inadequate nutrition education, particularly the emphasis on carbohydrate counting (CC) and the management of T1DM through a therapeutic patient education approach [4]. CC, in conjunction with insulin dosage adjustments, plays a pivotal role in the effective management of T1DM, improving metabolic control and enhancing the quality of life [5]. CC involves a dietary management strategy that quantifies the carbohydrate content of food, enabling individuals to administer the appropriate insulin dose to maintain optimal blood glucose levels [6]. The use of technology has proven effective in enhancing CC education and the overall learning experience [7,8]. To respond to the urgent demand to reach a balance in the treatment of T1DM, WebDia is an app designed to speed up CC by providing a complete list of foods, accompanied by their corresponding carbohydrate content [9]. The implementation of WebDia in Switzerland has yielded promising outcomes among individuals with T1DM, resulting in a reduction of glycated hemoglobin levels by 0.33% without a concurrent increase in hypoglycemia prevalence [10]. Moreover, users of WebDia have reported increased independence and self-confidence in managing their condition, leading to enhanced self-efficacy and improved self-management of T1DM [9].

Deficiencies in the health care system, such as the exclusive availability of specialized care at the tertiary level and insufficient training of health care workers (HCWs) in managing and diagnosing T1DM, impact the quality of life for individuals with this condition in LMICs, including Peru [3]. Peru’s health care system faces a number of challenges, including poor accessibility to information at different health care levels, insufficient financial resources, and a shortage of HCWs with adequate training in diabetes management [11]. Furthermore, the health care system’s response to individuals living with T1DM is notably deficient in terms of understanding the disease, and the information provided does not align with the unique needs and characteristics of this particular population [12]. Additionally, dietary patterns vary widely across different cultural and geographical contexts, influencing how people with T1DM approach CC. In Peru, the complexity of gastronomy, characterized by diverse ingredient combinations and traditional dishes, adds an additional challenge.

In this context, Peru finds itself in a favorable position to benefit from a tailored adaptation of WebDia, accompanied by the essential training of HCWs. The study had a dual purpose: first, to adapt WebDia to the Peruvian context as previously detailed, and second, to empower HCWs to enhance the quality of care they provide to individuals living with T1DM. Additionally, the study assessed the impact of WebDia-Mundi (new version of the Swiss app WebDia adapted to other geographic contexts) on the quality of life and glycated hemoglobin levels of children and adolescents living with T1DM.

## Methods

### Study Design

The study design was pre-post and usability study using mixed methods. The study used both qualitative and quantitative approaches across 3 regions of Peru with distinct demographic characteristics that contribute to their cultural and socioeconomic diversity (Arequipa, Lima, and Trujillo).

This study was carried out in 3 phases. The first phase involved adapting the WebDia app to the Peruvian context, spanning from March to December 2022. The second phase centered on training HCWs, children and adolescents living with T1DM, and their caregivers. A series of workshops was conducted to enhance diabetes care management, with a particular emphasis on refining skills related to CC. The third phase consisted of the evaluation of usability, knowledge acquisition, glycated hemoglobin levels, quality of life, perception of knowledge, and workshop satisfaction. Both phases were developed in March 2023. Furthermore, follow-up sessions were conducted 3 months later, from June to July 2023, to assess the sustainability of the acquired knowledge and skills.

### Participants

The study had different participants for each phase. The first phase (adaptation) involved a collaboration with a specialized dietitian and comprised individuals older than 18 years living with T1DM and caregivers of children with T1DM. In the second phase, HCWs responsible for managing individuals requiring insulin for diabetes were actively involved, alongside children and adolescents living with T1DM, 6 to 18 years old, and their corresponding caregiver. Subsequently, during the third phase (evaluation), all participants from the second phase were considered. Recruitment of participants across all 3 phases was conducted through a strategic social media outreach, using a snowballing technique to expand the participant pool.

### Study Procedures

#### First Phase: Adaptation

WebDia, an innovative mobile health (mHealth) app developed by JLM in response to his daughter’s diagnosis of T1DM, assists individuals with diabetes who use insulin to count carbohydrates and adjust insulin treatment [10]. Originally in French and tailored to the Swiss diet, the app required adaptation to suit the Peruvian context. One of the primary modifications was the translation of WebDia into Spanish, ensuring accessibility for most of the Peruvian population. Additionally, user-friendly features were adjusted, including redesigning the logo. The most critical adaptation involved integrating a food database that reflects the diversity of Peruvian cuisine, incorporating locally available foods, traditional dishes, and commonly consumed ingredients with culturally appropriate portion sizes.

For this purpose, the most prevalent foods consumed in Peru were identified by using food data from a previous CRONICAS project [13] and consulting a recipe book authored by a dietitian specializing in macronutrient counting. The selection of typical dishes was based on popular local foods from Lima, Arequipa, and Trujillo. However, we did not have access to a systematically compiled dataset of the most commonly consumed dishes by region. Instead, our selection was based on available data sources and expert consultation. Information on carbohydrate composition was then incorporated using different sources. For natural foods and traditional dishes, we used the Peruvian table of food composition [14] and values extracted from WebDia. For processed and ultraprocessed food, carbohydrates information was taken from the database developed by CRONICAS Center of Excellence in Chronic Disease at Universidad Peruana Cayetano Heredia, in collaboration with the University of North Carolina at Chapel Hill [13]. Additionally, although accessibility was considered in the app’s adaptation process, WebDia-Mundi is not currently designed to support multiple languages beyond Spanish.

This adaptation was then validated with a small group of participants, including people living with T1DM from different ages and their caregivers. The validation process consisted of collecting first impressions about the app, such as missing foods on the list, technical issues, and overall usability feedback. During this process, we also addressed specific needs to enhance accessibility, ensuring that the app could be used even by individuals who do not speak Spanish or who are illiterate. In the Peruvian version, we also incorporated multiple food photos to help users estimate food portions and make the weight of foods more visible. Based on this input, an improved version, now called WebDia-Mundi [15], was developed. Finally, a pilot usability study was conducted with the same group of participants, generating data that facilitated enhancements to various app functions, considering diverse perspectives (Figure S1 in Multimedia Appendix 1).

#### Second Phase: Training

Workshops were conducted across 3 regions of Peru (Arequipa, Lima, and Trujillo) targeting HCWs, children, and adolescents living with T1DM, and their caregivers (Table S1 in Multimedia Appendix 2). These workshops primarily focused on comprehensive training in CC, instructions on using the app to perform CC as part of self-monitoring, and how to adjust insulin doses in a personalized way.

For this purpose, the HCWs were involved in a 2-day face-to-face theoretical and practical training during which they lived like a person living with T1DM (they practiced doing CC and applying to themselves several insulin injections simulated with sodium chloride). HCWs were instructed to previously watch 3 short videos produced at Geneva University Hospitals (HUG) to lay the foundations for diabetes management using CC [16-18]. These instructional days were followed by a one-day practical workshop, bringing together HCWs, children and adolescents living with T1DM, and their caregivers. During this session, HCWs who were already acquainted with the WebDia-Mundi app collaborated with children and adolescents living with T1DM and their caregivers to provide guidance on CC and how to adapt insulin treatment. To ensure effective participation, participants were encouraged to download the app from either the Apple Store [19] or the Play Store [20] to ensure effective use during the session.

#### Third Phase: Evaluation

During the 2-day face-to-face session, a knowledge test was applied before and after the training. In addition, at the beginning of the third-day practical workshop, sociodemographic data, perceptions of knowledge, and quality of life (self-reported by the children and adolescents living with T1DM and the evaluation of them provided by their caregiver) were collected. If consent was provided by children and adolescents living with T1DM and their caregivers, a blood sample was collected for glycated hemoglobin analysis. At the end of the workshop, the usability of WebDia-Mundi and workshop satisfaction were assessed. Three months later, participants were recontacted for follow-up evaluations to assess the usability of WebDia-Mundi, perceptions of knowledge, quality of life, and glycated hemoglobin (specifically for children and adolescents living with T1DM).

### Outcomes

The primary outcome was the usability of WebDia-Mundi to explore the main issues in the app from the end users’ perspective. The usability assessment was carried out using the System Usability Scale (SUS) for children and adolescents living with T1DM and their caregivers [21]. This questionnaire is considered a reliable tool to measure the usability of products and services, including hardware, software, mobile devices, and apps. In addition, the questionnaires were supplemented with questions about perceived operability of the app, perceived ease of the app, satisfaction with the app, and open-ended questions about the participants’ experience of use. All questions were adapted and translated into Spanish using simple terminology to enhance comprehension.

Another primary outcome was enhancing knowledge of the HCWs. For this purpose, a pre and posttest was conducted at the beginning and at the end of the 2-day workshop sessions, to assess their knowledge. The test was developed by a diabetes educator. The questions were related to the topics taught during the training, such as diabetes, diet, CC, insulin adjustment, etc.

The following were predefined as secondary outcomes: glycated hemoglobin assessed by blood sampling using a validated clinical laboratory (SynLab ISO 9001:2015) in the 3 regions. Quality of life was measured using the Spanish validated PedsQL Diabetes Module (version 3.0; Mapi Research Trust) [22] using child self-report and parent proxy-report scales tailored to the respective age groups of the children. Perceptions of knowledge through specific questions. Workshop satisfaction was assessed by a questionnaire developed by the research team. To ensure accuracy and appropriateness, we carefully reviewed the Spanish version. Knowledge perception and workshop satisfaction were assessed using specific questions developed by the research team (Table S5 in Multimedia Appendix 2).

### Data Evaluation

#### The SUS

The SUS was administered to all participants, using a Likert scale ranging from 1 to 5, where 1 indicates strongly disagree, and 5 indicates strongly agree. For the analysis, we used the standard approach to generate a final score ranging from 0 to 100. To do so, we converted the scores with a different setting for odd and even items (respectively, positive and negative tone items). In addition, if a respondent leaves an item blank, it is assigned a raw score of 3 (the center of the 5-point scale). The adjusted scores were then summed and multiplied by 2.5 to obtain the standard SUS score [21].

#### Knowledge Test (Pre-Post)

A pre-post knowledge test was administered to HCWs, and the final score, ranging from 0 to 20, was calculated by adding the individual scores for each question. For more details on the content and level of the test, please refer to the questions in Table S5 in Multimedia Appendix 2.

#### Glycated Hemoglobin

The assessment of the glycated hemoglobin test was conducted in children and adolescents living with T1DM. The results were obtained from blood samples analyzed in a laboratory that had been validated across all 3 regions.

#### Quality of Life

The PedsQL 3.0 Diabetes was used to assess the quality of life in children and adolescents living with T1DM, considering perspectives from both children and adolescents living with T1DM and their caregivers. Like the SUS, a Likert scale (0‐4) was used across five domains: (1) diabetes symptoms, (2) treatment I, (3) treatment II, (4) worry, and (5) communication. Treatment I is used to indicate the presence or absence of a problem, while Treatment II is used to measure the degree of difficulty associated with the problems identified. The items were then reverse transformed to a scale of 0‐100 (0=100, 1=75, 2=50, 3=25, 4=0), with higher scores indicating better quality of life. If a respondent left an item blank, it was assigned a score of 2 (the center of the 5-point scale) [22].

#### Perception of Knowledge

Knowledge perception questions were assessed for all participants, using a knowledge rating scale from 1 to 5, with 5 representing the highest score.

#### Workshop Satisfaction

Satisfaction with the workshops was evaluated for all participants by extracting the most recurrent phrases from the open-ended questions.

### Statistical Methods

Data analysis was conducted in STATA for Windows (version 17; StataCorp). The description of the study population was carried out using absolute and relative frequencies for categorical variables, while numerical variables were summarized using the median and the 25th and 75th percentiles. To assess changes in quality of life, perceived knowledge, and usability as reported by children and adolescents living with T1DM and their caregivers, the Wilcoxon matched pairs test and McNemar’s chi-square test were used. A *P* value <.05 was considered significant. In addition, we used line graphs to visualize the progression of acquired knowledge before and after the training workshop, as well as to illustrate changes in glycated hemoglobin levels at the 3-month mark.

### Ethical Considerations

The study was approved by the Institutional Review Board of Universidad Peruana Cayetano Heredia (approval number 513-35-22, registration code 208867). All participants provided their consent, with the option to withdraw at any time. Given that the study population was from urban areas and no specific cultural adaptations were required, the main consideration was ensuring that the language used in the consent form was clear and easily understood by participants. For children younger than 18 years, assent was obtained after a detailed explanation of the study’s objectives, benefits, and risks. During the workshop phase, consent from only one caregiver was required. In cases where the child was below 6 years old, both the child and the caregiver had the option to participate in the activities; however, the data collected from this group were excluded from the analysis. All collected data were deidentified, and only the first authors had access to safeguard participant information. While no direct financial compensation was provided, participants received support for travel, accommodation, and meals as needed, particularly for those coming from regions outside the study site.

## Results

### Demographic Characteristics

Participants for all study phases were selected using a convenience sampling approach, based on their availability and willingness to participate. A total of 25 HCWs and children and adolescents living with T1DM, along with their caregivers, were initially enrolled. Subsequently, 24 HCWs and 19 children and adolescents living with T1DM and their caregiver were included in the study. For the follow-up phase, 12 children and adolescents living with T1DM and their caregivers were retained in the study (Figures S2 to S4 in Multimedia Appendices 3-5). Of the total HCWs, 18 (75%) were female. They reported caring for a diverse range of diabetes patients, with a median of 50 (IQR 5-150) patients with T2DM and a median of 5 (IQR 2-12) patients with T1DM. The perceived knowledge of T1DM was 12 (50%) fair and 9 (38%) good, perceived knowledge of CC was rated as 6 (25%) poor and 11 (46%) fair, and perceived knowledge of insulin therapy was reported as 11 (46%) poor and 7 (29%) good (Table 1).

Regarding children and adolescents living with T1DM, the median age was 12 (IQR 10‐16) years, and their caregiver had a median age of 47 (IQR 40‐51) years, with 15 (79%) caregivers being female. The primary caregiver of children and adolescents living with T1DM included other family members in addition to parents, but most of the attendees were mothers. Around 13 (72%) caregivers had a university or technical education. In terms of perceived knowledge, 13 (68%) caregivers had a fair perception of T1DM, and 4 (21%) caregivers considered it good. For CC, 7 (37%) caregivers rated their knowledge as poor, and 6 (32%) caregivers rated it as fair, while for insulin therapy, 8 (42%) caregivers considered it fair, and 8 (42%) caregivers considered it good (Table 2).

**Table 1.** Sociodemographic characteristics of participants.

Health care workers (n=24)	Values
Age, median (P25-P75)	48 (41-54)
Sex^a^, n (%)	
Female	18 (75)
Male	5 (21)
Profession^a^, n (%)	
Pediatric endocrinologist	3 (13)
Adult endocrinologist	2 (8)
Medicine	6 (25)
Nursing	7 (29)
Nutrition	3 (13)
Pharmacist	1 (4)
Diabetes educator^a^, n (%)	
Yes	10 (42)
No	13 (54)
T1DM knowledge^a^, n (%)	
Very good	1 (4)
Good	14 (58)
Fair	8 (33)
Poor	0 (0)
Very poor	0 (0)
T1DM^b^ knowledge^a^, n (%)	
Very good	0 (0)
Good	9 (38)
Fair	12 (50)
Poor	2 (8)
Very poor	0 (0)
Carbohydrate counting knowledge^a^, n (%)	
Very good	0 (0)
Good	5 (21)
Fair	11 (46)
Poor	6 (25)
Very poor	1 (4)
Insulin therapy knowledge^a^, n (%)	
Very good	3 (13)
Good	7 (29)
Fair	11 (46)
Poor	1 (4)
Very poor	1 (4)

a Results may not add due to missing values.

b T1DM: type 1 diabetes mellitus.

**Table 2.** Sociodemographic characteristics of children and adolescents living with type 1 diabetes mellitus and their caregiver/parent.

Characteristics	Values
Children living with T1DM^a^ (n=19)	
Age, median (P25-P75)	12 (10-16)
Sex, n (%)	
Female	10 (53)
Male	9 (47)
Time since T1DM^a^ diagnosis (months), median (P25-P75)	36 (15-56)
Type of insurance^b^, n (%)	
Integrated Health Insurance	5 (28)
Social Security (EsSalud)	9 (50)
Private	4 (22)
Chronic complications^b^, n (%)	
Yes	2 (11)
No	16 (89)
Type of insulin treatment^b^, n (%)	
Human insulin	2 (11)
Analog insulin	16 (89)
Glucometer use, n (%)	
>5 times per day	7 (37)
2‐5 times per day	7 (37)
Once a day	3 (16)
<1time per day	1 (5)
Do not use	1 (5)
Use of continuous glucose monitor^b^, n (%)	
Yes	8 (44)
No	10 (56)
Medical visits in the past 12 months, median (P25-P75)	4 (1-6)
Medical visits in the past 3 months, median (P25-P75)	1 (0‐2)
Hospitalizations in the last 12 months, median (P25-P75)	0 (0‐1)
Hospitalizations in the last 3 months, median (P25-P75)	0 (0‐0)
Severe hypoglycemia in the last 12 months^b^, median (P25-P75)	1 (0‐4)
Severe hypoglycemia in the last 3 months, median (P25-P75)	0 (0‐2)
Glycated hemoglobin (%), median (P25-P75)	7.1 (5.7‐8.0)
Carbohydrate counting knowledge^b^, n (%)	
Very good	0 (0)
Good	1 (6)
Fair	11 (61)
Poor	3 (17)
Very poor	3 (17)
Parents/caregivers (n=19)	
Age, median (P25-P75)	47 (40-51)
Sex^b^, n (%)	
Female	15 (79)
Male	3 (16)
Highest level of education^b^, n (%)	
Primary incomplete	1 (5)
Secondary complete	4 (21)
Technical	5 (26)
University	8 (46)
Relationship with the person with T1DM^a^ ^b^, n (%)	
Mother	16 (84)
Father	1 (5)
Aunt/Uncle	1 (5)
T1DM^a^ knowledge^b^, n (%)	
Very good	0 (0)
Good	4 (21)
Fair	13 (68)
Poor	1 (5)
Very poor	0 (0)
Carbohydrate counting knowledge^b^	
Very good	0 (0)
Good	2 (10)
Fair	6 (32)
Poor	7 (37)
Very poor	3 (16)
Insulin therapy knowledge^b^	
Very good	1 (5)
Good	8 (42)
Fair	8 (42)
Poor	1 (5)
Very poor	0 (0)

a T1DM: type 1 diabetes mellitus.

b Results may not add due to missing values.

### WebDia-Mundi Usability

HCWs achieved a median overall usability score of 76%, reflecting positive results for perceived operability of the app and partially positive findings for perceived app, ease of the app, and satisfaction with the app (Table S2 in Multimedia Appendix 2).

HCWs also highlighted the practicality of CC in the diet as follows:

*Very useful in our country, where carbohydrates are prevalent, and misconceptions about their restriction in diabetes cases are common.*

*It’s user-friendly, allows saving calculation data, and the dish photos are highly beneficial.*

*It fosters creativity, letting me combine available ingredients to meet carbohydrate limits.*

On the other hand, the app recorded an overall usability score of approximately 81% for children and adolescents living with T1DM, which significantly dropped to a median of 57% at 3 months. Likewise, caregivers had an initial median score of 74% during the workshop, which decreased significantly to 54% at 3 months. Notably, positive results were observed for perceived operability of the app, perceived ease of the app, and satisfaction with the app in both groups, with no significant changes noted at the 3-month mark (Table 3).

Children and adolescents living with T1DM and their caregivers consistently emphasized the app’s advantages. Statements from children and adolescents living with T1DM included:

*It helps to calculate carbohydrates more accurately.*

*It improves control of quantities at mealtimes.*

*It promotes independence, gradually building knowledge in carb counting.*

Moreover, they expressed that they found the app highly useful and user-friendly. In parallel, caregivers mentioned their perspectives with comments such as:

*It makes it easier for me to understand carbohydrate amounts for my children.*

*It has the potential to prevent hypoglycemia and hyperglycemia.*

*I can refine carbohydrate counting for enhanced control.*

*It increases my confidence in accurately calculating carbohydrates.*

**Table 3.** Usability of WebDia reported by the children and by their parent/caregiver at baseline and 3 months later.

Characteristics	Baseline	3 months follow-up	*P* value
Children living with T1DM^a^			
Overall score, median (P25-P75)^b^	80.8 (51.9‐85.6)	56.7 (3.8‐71.2)	.02
Perceived operability of the app			
I could use it by myself^c^			.10
Yes	11 (58)	6 (50)	
No	3 (16)	5 (42)	
I believe that offers benefits^c^			.32
Yes	14 (74)	10 (83)	
No	0 (0)	1 (8)	
Perceived ease of the app			
Difficulties using the app^c^			.32
Yes	0 (0)	5 (42)	
No	14 (74)	5 (42)	
Difficulties with carbohydrate counting^c^			.32
Yes	4 (21)	5 (42)	
No	11 (58)	6 (50)	
Satisfaction with the app			
Instructions were adequate^c^			.32
Yes	15 (79)	10 (83)	
No	0 (0)	1 (8)	
Recommended app^c^			.32
Yes	15 (79)	10 (83)	
I don’t know	0 (0)	1 (8)	
Parents/caregivers			
Overall score, median (P25-P75)^b^	73.6 (51.9‐85.6)	54.3 (18.3‐68.8)	.03
Perceived operability of the app			
I could use it by myself^c^			.32
Yes	14 (74)	7 (58)	
No	4 (21)	5 (42)	
I believe that offers benefits^c^			>.99
Yes	18 (95)	11 (92)	
No	0 (0)	1 (8)	
Perceived ease of the app			
Difficulties using the app^c^			.03
Yes	1 (5)	7 (58)	
No	17 (90)	5 (42)	
Difficulty of carbohydrate counting^c^			.71
Yes	8 (42)	5 (42)	
No	10 (53)	6 (50)	
Satisfaction with the app			
Instructions were adequate^c^			>.99
Yes	18 (95)	11 (92)	
No	0 (0)	1 (8)	
Recommended app^c^			>.99
Yes	18 (95)	11 (92)	
I don’t know	0 (0)	1 (8)	

a T1DM: type 1 diabetes mellitus.

b Wilcoxon matched pairs test was used.

c McNemar chi-square test was used. Results may not add due to missing values.

Common drawbacks identified during the workshop included the absence of specific dishes and foods, the necessity for adaptations tailored to different cities, the significance of differentiating between rapidly absorbed and complex carbohydrates, the demand for more detailed information regarding individual meal ingredients, and the desire to compare multiple meal options simultaneously based on their carbohydrate content. Furthermore, technology access was acknowledged as a potential limitation, particularly for individuals residing in areas with limited internet or smartphone connectivity. However, both children and adolescents living with T1DM and their caregivers expressed a strong motivation to frequently use WebDia-Mundi.

### Knowledge Obtained in the Workshops

#### Overview

**Figure 1.**
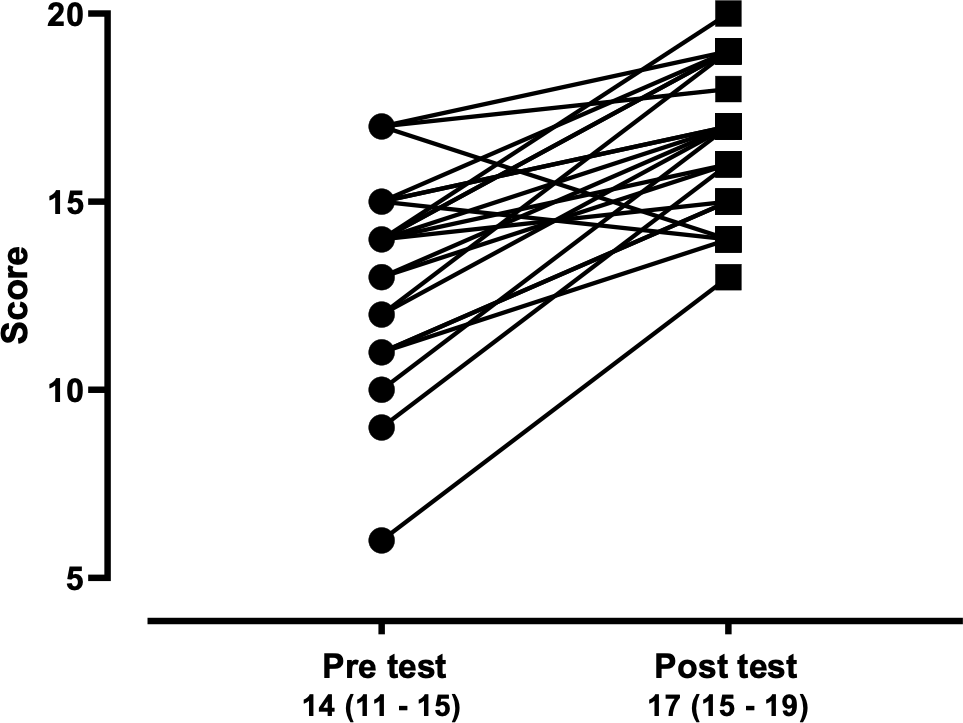
Pre- and posttest scores from health care workers (n=23) attending the workshops.

The HCW pretest scores ranged from 6 to 17, with a median of 14.0 (IQR 11.5‐15.0), while the posttest scores ranged from 13 to 20, with a median of 17.0 (IQR 15.0‐19.0). There was a significant increase between pre- and posttest scores (*P*=.001, Figure 1).

#### Glycated Hemoglobin

**Figure 2.**
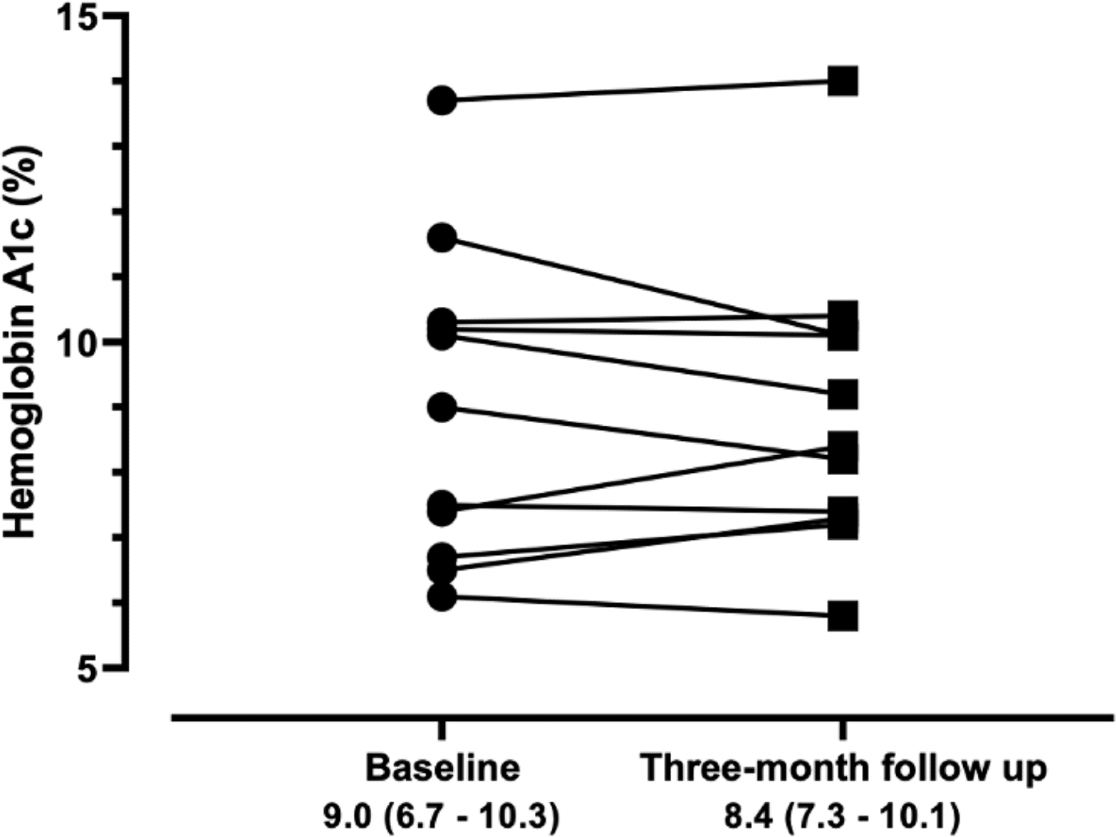
Comparison of hemoglobin A_1c_ levels in participating children during the workshop and at the 3-month follow-up.

Glycated hemoglobin levels were measured in children and adolescents living with T1DM at the workshop and 3 months later. On the workshop day, the glycated hemoglobin level was 9.0% (IQR 6.7%‐10.3%), ranging from 5.4% to 13.7%. At the 3-month follow-up, the glycated hemoglobin level was 8.4% (IQR 7.3%‐10.1%), ranging from 5.8% to 14.0%, without changes in comparison to the baseline (*P*=.789, Figure 2).

#### Quality of Life

Across all domains, scores were close to 60, with a slight increase in worry and a marginal decrease in diabetes symptoms, communication, and treatment-related aspects. The overall score showed a slight increase for both, but the changes observed from baseline to follow-up were not statistically significant (Table 4). On the other hand, no significant differences were found between the perception provided by children and adolescents living with T1DM and their caregivers (Table 3 in Multimedia Appendix 2).

**Table 4.** Quality of life reported by the children and by their parent/caregiver at baseline and 3 months later. Wilcoxon matched pairs test was used.

Characteristics	Baseline	3 months follow-up	*P* value
Children living with T1DM^a^ (P25-P75)			
Diabetes symptoms	59.1 (56.8‐77.3)	54.6 (50.0‐72.7)	.35
Treatment I	56.3 (50.0‐81.3)	50.0 (50.0‐75.0)	.44
Treatment II	67.9 (50.0‐82.1)	53.6 (50.0‐78.6)	.27
Worry	41.7 (25.0‐50.0)	50.0 (50.0‐50.0)	.20
Communication	66.7 (33.3‐91.7)	50.0 (50.0‐75.0)	.95
Overall	60.5 (43.9‐69.7)	61.0 (52.3‐70.9)	.16
Parents/caregivers, median (P25-P75)			
Diabetes symptoms	65.9 (50.0‐75.0)	50.0 (50.0‐59.1)	.08
Treatment I	50.0 (37.5‐68.8)	50.0 (50.0‐75.0)	.56
Treatment II	60.7 (46.4‐78.6)	50.0 (50.0‐75.0)	.52
Worry	41.7 (33.3‐50.0)	50.0 (50.0‐58.3)	.11
Communication	54.2 (16.7‐83.3)	50.0 (50.0‐75.0)	.40
Overall	56.9 (42.6‐68.9)	62.4 (56.4‐75.8)	.11

a T1DM: type 1 diabetes mellitus.

### Perception of knowledge

The perception of CC and T1DM knowledge, when compared with 3 months after the workshop, showed a significant decrease in median reported knowledge among both children and adolescents living with T1DM and their caregivers (Table 5).

**Table 5.** Difference in knowledge at 3-month follow-up in children living with type 1 diabetes mellitus and their caregivers. Wilcoxon matched pairs test was used.

Characteristics	Baseline	3 months follow-up	*P* value
Children living with T1DM^a^, median (P25-P75)			
Carbohydrate counting knowledge	3 (3-4)	3 (2-3)	.03
Parents/caregivers, median (P25-P75)			
T1DM^a^ knowledge	3 (3-3)	2 (2-3)	.02
Carbohydrate counting knowledge	3 (3-4)	3 (2-4)	.04
Insulin therapy knowledge	2 (2-3)	2 (2-2)	.75

a T1DM: type 1 diabetes mellitus.

### Workshop Satisfaction

HCWs found that the workshops not only taught them about CC and the use of WebDia-Mundi but also allowed them to see T1DM from the perspective of children and adolescents living with T1DM and their caregivers. They expressed an understanding that there is no need to impose food restrictions when managing T1DM, and those with T1DM can lead normal lives if they practice CC. Some HCWs mentioned the following:

*No need for prohibitions or excessive restrictions.*

*Type 1 diabetics can lead a normal life.*

Regarding changes they plan to make in their practice after the workshop, HCWs expressed a commitment to active listening, considering patients’ contexts, and practicing empathy. They mentioned intentions such as:

*Listen and provide more support.*

*Empathize and put myself in their shoes.*

*Help caregivers understand it’s not their fault, is a manageable condition.*

*Show greater interest in their well-being and challenges.*

*Empower them to manage the condition and live a normal life.*

*Always prioritize the patient’s feelings.*

HCWs found the most positive aspect of the workshop to be the valuable interactions with children and adolescents living with T1DM and their caregivers. On the other hand, the most negative aspect, as reported by HCWs, was the discomfort of self-administering glucose measurements and injections during the practical sessions. Their feedback included:

*The hands-on experience was enriching, allowing us to experience it ourselves, administering NaCl and measuring glucose. It was a unique and fascinating practical course in my professional life.*

*Living as a diabetic [person living with diabetes], understanding the effort required to measure glucose, calculate meals, and make injections, highlighted the time and dedication patients invest in normalising their glucose.*

*Practising self-monitoring of glucose and experiencing it first-hand truly deepened our understanding of the patient’s perspective.*

Finally, the comments and reflections of the HCWs underscored the workshop’s significant influence on their perspectives and professional approaches. They emphasized the need to expand participation, sharing this knowledge with professionals from various health care organizations, expressing interest in specialized workshops, and proposing the creation of camps and activities for children and adolescents living with T1DM.

The caregiver also showed significant interest in the workshop. Most of them found the workshop duration suitable, and all reported valuable knowledge gained, resulting in a positive learning outcome. Their feedback emphasized the importance of CC and the usefulness of CC apps. Some comments mentioned were:

*I gained a better understanding of carbohydrates.*

*The carbohydrate counting app proved to be incredibly useful.*

*Maintaining a positive outlook is crucial; it helps build confidence that things happen for a reason.*

In summary, the lifestyle changes considered by the children and adolescents living with T1DM and their caregivers were predominantly centered around dietary choices and the use of WebDia-Mundi (Table S4 in Multimedia Appendix 2).

## Discussion

### Principal Results

Our findings indicate a favorable response to the WebDia-Mundi app (an adaptation of the WebDia app tailored to the Peruvian context) among HCWs, children and adolescents living with T1DM, and their caregivers. The incorporation of popular local foods and an easy-to-use interface were key factors in ensuring the app’s effective adaptation in Peru. Additionally, the app played an important role in enhancing users’ understanding of CC, which is essential for better nutritional management and quality of life. The training provided to HCWs improved their knowledge regarding the care they deliver to individuals with T1DM. It is important to note that most HCWs are more familiar and comfortable providing dietary advice for individuals with type 2 diabetes and often feel less confident when counseling patients with T1DM. Moreover, children and adolescents living with T1DM, and their caregivers, exhibited a generally positive perception of their quality of life. While the use of WebDia-Mundi resulted in slight improvements in specific domains, these enhancements did not reach statistical significance. Furthermore, there were no notable changes observed in the levels of glycosylated hemoglobin among children and adolescents living with T1DM. In the management of T1DM in LMICs, having tools that alleviate the burden of self-managing glucose levels is crucial. One of the key challenges faced by people living with T1DM is understanding how to manage their meals effectively. Traditionally, many individuals with T1DM followed highly restrictive dietary patterns due to limited knowledge about CC. Providing an accessible and practical app empowers both patients and caregivers, while improving HCWs’ understanding of CC contributes to better overall care.

### Comparison With Prior Work

The general knowledge was low among the participants of the workshop. Other studies with physicians and nurses show similar results on the inadequate training in the treatment of T1DM [23-25]. A parallel scenario emerged among children and adolescents living with T1DM and their caregivers, highlighting a perceived inadequate knowledge concerning self-management and dietary practices [26]. It is noteworthy that enhancing the knowledge of CC has been proven to positively impact the self-management of the disease [27]. Nevertheless, 3 months into the training, there was a notable decline in the perceived knowledge regarding CC and T1DM. This decline could be attributed to diverse postintervention factors that might have adversely impacted self-perceived knowledge when attempting to implement the acquired skills from the workshops, especially in the practical application of CC in daily life, or they discovered there was much more to learn than they initially thought as they underwent the training [28,29].

Regarding the usability of WebDia-Mundi, HCWs expressed a positive attitude towards operability, ease, and satisfaction, emphasizing the practicality of the CC in the diet and the promotion of an unrestricted diet. Similarly, both children and adolescents living with T1DM and their caregivers initially shared a similar attitude. However, over the course of 3 months, this positive attitude decreased significantly by 24 and 20 percentage points for children and adolescents living with T1DM and their caregivers, respectively, resulting in an attitude close to neutral. Despite this change in attitude, there were no statistically significant differences observed in perceived operability, satisfaction, and ease. The lack of significance in these measures may be attributed to the small sample size, as numerical values are more vulnerable to minor fluctuations compared with relative frequency. Additionally, the observed lack of significance may be linked to a disinterest in using the app, given that a low proportion of adolescents have been reported to adhere to the use of such apps [30]. The mHealth adherence rate averages 56%, highlighting the necessity for enhancement through customization options and reminder notifications for improved usability [31].

Furthermore, our results show no significant changes in the mean glycosylated hemoglobin level of children and adolescents living with T1DM after 3 months. These findings are consistent with previous reports suggesting that while changes have been observed with CC support apps at 3 months in adults, no similar changes were found in the pediatric age group, possibly due to the inherent difficulty in controlling diet in this population [32,33]. It is essential to note that the patient management approach in Peru exhibited clear restrictions, suggesting that introducing an alternative management style might necessitate an adaptation period for patients to assimilate the concept of CC, potentially extending beyond 3 months. While certain apps, such as the Swiss version (ie, WebDia), have explored the option of displaying insulin amounts to enhance user-friendliness [10,34], this was not implemented in Peru due to regulatory requirements and the need for supervised implementation by trained HCWs to mitigate potential clinical consequences [35]. Furthermore, there was limited awareness among patients’ treating physicians about WebDia-Mundi, and the research team did not provide close follow-up.

Most existing mHealth apps for CC are free; however, their food databases are often generic, lacking cultural specificity [36,37]. The complexity of CC varies significantly depending on the dietary context of each country. One widely recommended feature in previous studies is the use of visual food representations, which was also preferred by participants in our validation process [38]. As a result, we incorporated more visual photos to enhance usability. While the original Swiss version included only 20 food images, the Peruvian version was enriched with 108 photos to better support users in estimating portion sizes and improve the app’s practical utility. Furthermore, previous studies have not been conducted in LMICs, where challenges such as limited access to health care and medications can significantly impact outcomes related to quality-of-life improvements for people living with T1DM. However, despite the lack of changes in glycosylated hemoglobin, participants reported improvements in the management and control of their disease, citing increased self-confidence [39]. Some participants mentioned feeling more freedom in their dietary choices and greater confidence when deciding what to eat. Additionally, children were able to assist their caregivers and became more aware of CC, making the process less stressful for both. Consequently, our results on the perception of the quality of life of both children and adolescents living with T1DM and their caregivers, although not statistically significant, revealed a slight increase in the overall score.

A frequently suggested enhancement for WebDia-Mundi involves expanding the information provided for each preparation or food, such as displaying ingredients considered, carbohydrate typology, and comparisons with similar preparations. While this could enhance user decision-making, there’s a potential trade-off, as it might increase the complexity of the app [34]. Access to technology also surfaced as a potential limitation, particularly for individuals residing in areas with restricted internet or smartphone connectivity. This is one of the most common challenges encountered in the implementation of digital health services or mHealth solutions [40].

Regarding the workshop, the results indicate a noteworthy increase in knowledge acquisition among HCWs. Beyond demonstrating improved understanding of CC and the use of WebDia-Mundi, they also expressed gaining insights into the lived experience of an individual living with T1DM. These workshops adhere to the basic education outlined in the principles of the National Assessment Framework, which is derived from the National Diabetes Services Plan in Australia [41]. This educational approach aims to advance knowledge and understanding, but to achieve significant behavioral changes in HCWs, it is essential to implement a comprehensive training program [41]. Similarly, children and adolescents living with T1DM and their caregivers provided positive feedback about the workshop, citing the acquisition of valuable knowledge, particularly in the realm of making informed food choices. The involvement of children and adolescents living with T1DM and their caregivers in these workshops facilitates a thorough assessment of the needs and expectations for enhancing educational programs tailored for individuals living with T1DM [42].

### Public Health and Clinical Relevance

The adapted app, WebDia-Mundi, holds the potential to enhance access to diabetes management tools, especially in LMICs. By offering an adaptable tool for diverse cultural settings, it could contribute to reducing disparities in health care and improving equity in access to quality resources for T1DM control [40]. Additionally, the app can serve as a valuable tool for health promotion and education, as the integration of mHealth and health coaching for individuals with chronic diseases has demonstrated a positive impact on both the quality of life and self-management of the disease [43]. Facilitating CC and insulin management can empower individuals living with T1DM and their caregivers to make informed decisions about their treatment and enhance self-care, regardless of their location. However, the adaptation process posed significant challenges, highlighting the need for more agile and scalable approaches to effectively adapt mHealth tools to different countries. Cultural and linguistic differences, as well as variations in dietary habits and health systems, require a structured but flexible adaptation framework. Multistakeholder involvement was essential to ensure that the tool meets the needs of the target population. In addition, e-learning strategies could facilitate the implementation of WebDia-Mundi, improving its accessibility and ease of use for both HCWs and end users in a variety of settings. On the clinical side, WebDia-Mundi could be an effective resource for HCWs in clinical T1DM management by simplifying carbohydrate intake tracking and insulin dosing, ensuring that CC education remains accessible to a wide range of users, including those who are illiterate or speak a native language other than Spanish [44]. Furthermore, WebDia Mundi might be beneficial for caregivers by enabling them to better understand diabetes management and providing practical tools to support patients more effectively [10].

### Limitations

This study has several strengths that significantly contribute to its impact and reliability. First, the successful adaptation of the WebDia app to the Peruvian context highlights the adaptability of WebDia-Mundi. Its potential to address and integrate diverse dietary and cultural needs highlights the possibility of scalability to other countries with no costs for the users. Second, the comprehensive approach, incorporating both quantitative and qualitative methodologies, offers a more complete assessment of the app’s effectiveness. Additionally, the active involvement and collaboration among HCWs, children and adolescents living with T1DM, and their caregivers throughout the study phases ensure validity and reflect a strong commitment to enhancing T1DM care within the Peruvian context. It is important to highlight that the collaboration among dietitians, endocrinologists, app developers, and the diabetes community throughout the study phases ensured that the adaptation process was user-centered and aligned with the real needs of the target population. The participatory approach ensured that WebDia-Mundi met the dietary and cultural needs of the target population. Furthermore, the inclusion of 3 distinct cities in Peru, each with its unique characteristics, although not exhaustive of Peru’s geographical diversity, enhances the heterogeneity of our findings. However, it is important to acknowledge certain limitations. The absence of a control group in specific aspects of the study restricts direct comparisons with individuals who did not participate in the research. Additionally, the short follow-up period may have limited our ability to observe significant changes in HbA_1c_ and long-term behavioral adaptations. Another key limitation is the potential bias inherent in using a convenience and snowball sampling method and recruiting participants through social media. This approach may have inadvertently led to the inclusion of individuals already inclined towards undertaking the course due to their existing interest, potentially skewing our sample towards those with lower levels of knowledge. The lack of heterogeneity in education levels among caregivers may have influenced the feedback on the app’s usability and accessibility, leading to different perspectives on its ease of use and practical applicability. Furthermore, this recruitment strategy could impact the external validity of our findings. However, it is important to highlight that recruiting people with T1DM presents significant challenges, even when using traditional recruitment methods. Based on our experience in patient recruitment, social media and community networks have proven to be among the most effective ways to engage participants in this context. Nevertheless, it is crucial to emphasize that this study serves as a pilot project, not intended to yield universally applicable results. Moreover, a technical barrier in LMICs is the potential existence of areas with lower mobile literacy levels or limited internet access. This might prevent widespread and equitable use of app-based tools, possibly excluding certain populations from fully benefiting from technological health solutions [45]. Finally, the initial sample size was not reached, and therefore, the power is lower, or the ability to detect small changes is lower.

### Conclusion

WebDia-Mundi is a tool for enhancing the self-management of people living with T1DM, covering both children and adolescents. Although clinically significant improvements in glycosylated hemoglobin have not been achieved, the application has received favorable acceptance and demonstrated efficacy in increasing dietary education, enabling a life free from unnecessary restrictions. It is crucial to acknowledge that additional efforts are necessary to enhance implementation, which requires further pretesting. Nonetheless, this study serves as an example of how tailoring mHealth solutions to the specific context of LMICs can contribute significantly to enhancing the quality of life for individuals with T1DM. Future research is needed to assess the long-term impact of the app and explore its adaptation to other cultural settings and evaluate strategies to reach a more diverse population, including people with different languages, literacy levels, and educational backgrounds, to ensure equitable access and ease of use.

## Supplementary material

10.2196/58029 Multimedia Appendix 1 Process flowchart for adapting WebDia to the Peruvian context and create WebDia - Mundi.

10.2196/58029 Multimedia Appendix 2 Overview of session design, WebDia Mundi usability, quality of life outcomes, workshop satisfaction, and knowledge perception.

10.2196/58029 Multimedia Appendix 3 Flowchart of health care workers attending the workshops by study site.

10.2196/58029 Multimedia Appendix 4 Flowchart of children living with type 1 diabetes mellitus attending the workshops by study site.

10.2196/58029 Multimedia Appendix 5 Flowchart of caregivers/parents of individuals living with type 1 diabetes mellitus attending the workshops by study site.

## Abbreviations

CC: carbohydrate counting
HCW: health care worker
HUG: Geneva University Hospital
LMIC: low- and middle-income country
mHealth: mobile health
SUS: System Usability Scale
T1DM: type 1 diabetes mellitus
