# Supplementary figures and images for: Adaptation of an mHealth Solution for the Nutritional Management of Diabetes in a Low- and Middle-Income Country: Pre-Post Mixed Methods Pilot Study
Source: JMIR Mhealth Uhealth. 2025 Sep 25;13:e58029. doi: 10.2196/58029 (PMC12463340; doi:10.2196/58029)

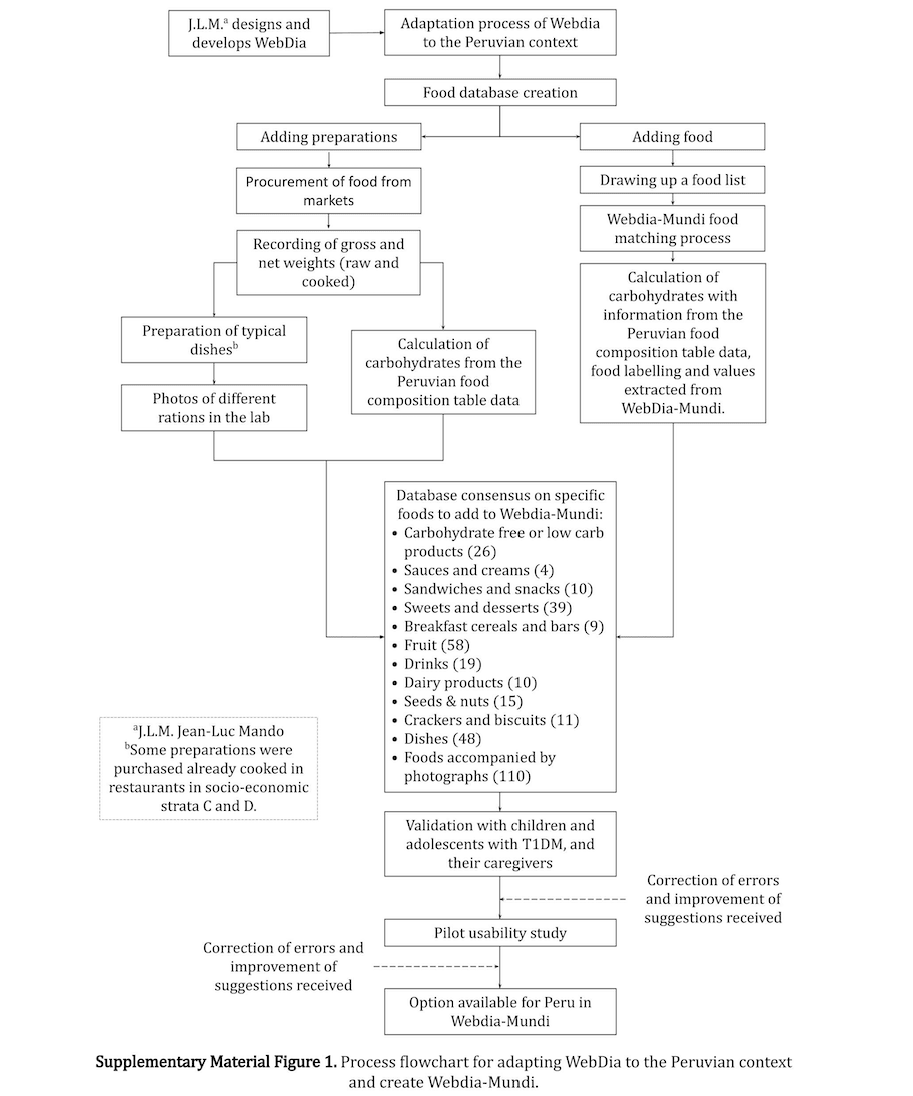

Supplement: Multimedia Appendix 1 [file mhealth-v13-e58029-s001.png]

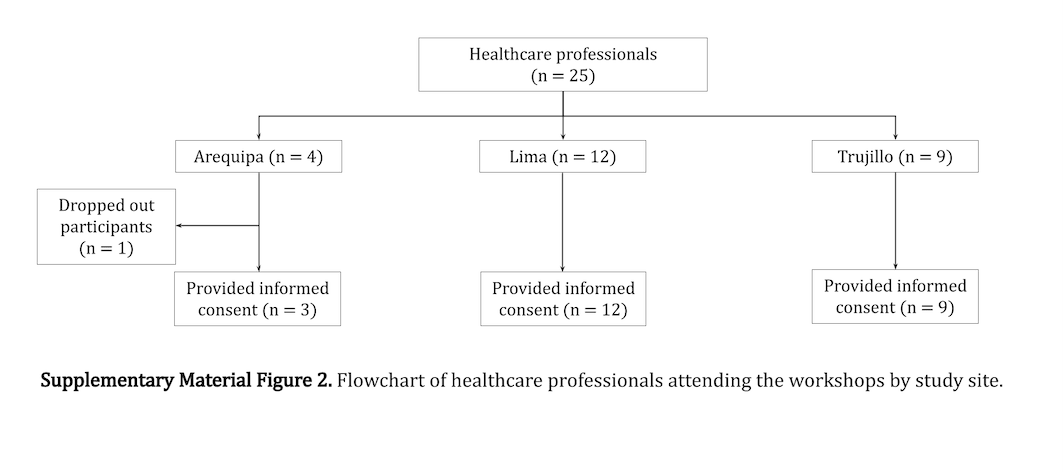

Supplement: Multimedia Appendix 3 [file mhealth-v13-e58029-s003.png]

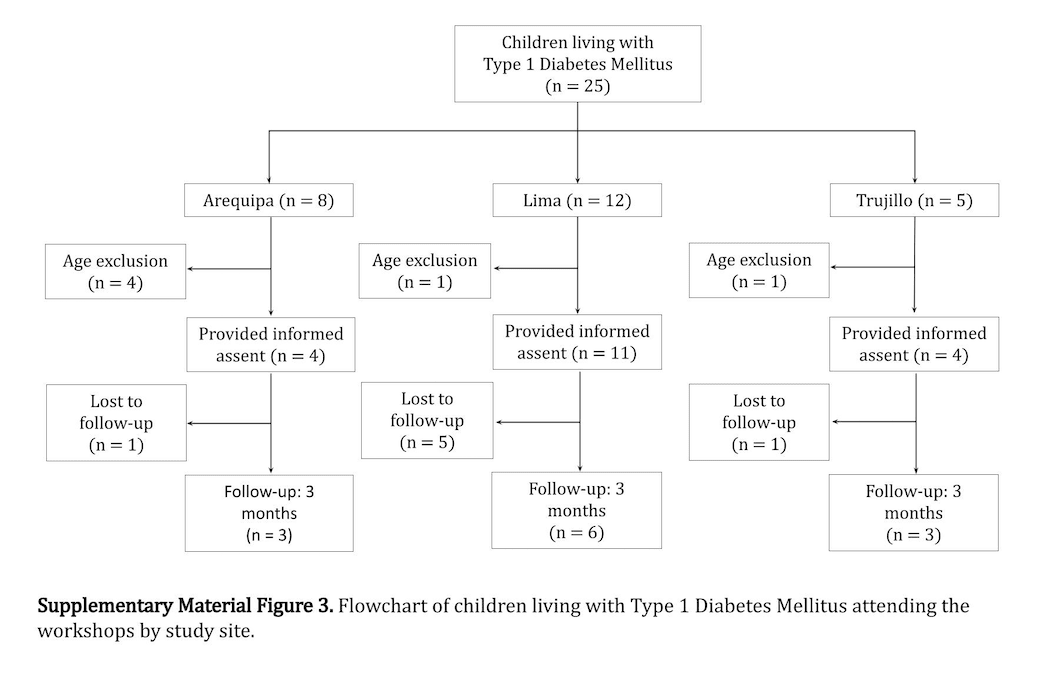

Supplement: Multimedia Appendix 4 [file mhealth-v13-e58029-s004.png]

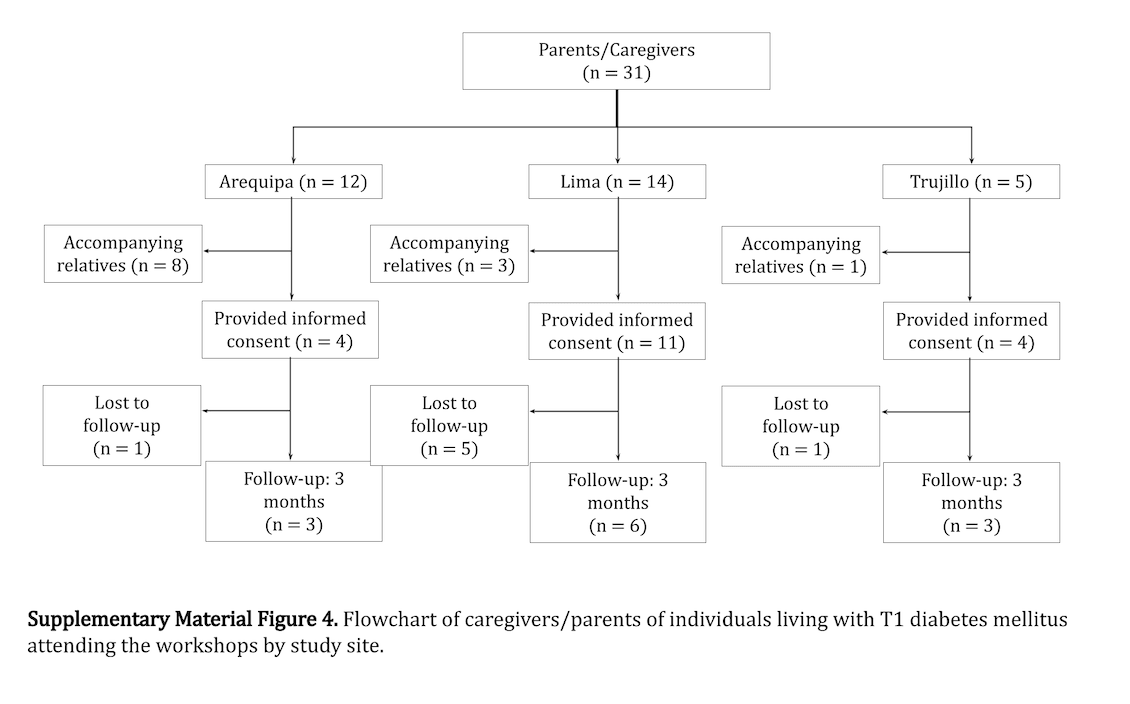

Supplement: Multimedia Appendix 5 [file mhealth-v13-e58029-s005.png]
