# Supplementary material for: Adaptation of an mHealth Solution for the Nutritional Management of Diabetes in a Low- and Middle-Income Country: Pre-Post Mixed Methods Pilot Study
Source: JMIR Mhealth Uhealth. 2025 Sep 25;13:e58029. doi: 10.2196/58029 (PMC12463340; doi:10.2196/58029)
Supplement: Multimedia Appendix 2 [file mhealth-v13-e58029-s002.docx]

**Supplementary Material**

**Table S1.** Educational session plan

| Theme | Activity | Duration (minutes) |
| --- | --- | --- |
| **DAY 1** | | |
| Welcome | Physical activity with music | 30 |
| Presentation | Introduction round – everyone shares their name, training objective, and hobby | 30 |
| Pre-Test | | 30 |
| Living as a Person with Diabetes | Discussing personal challenges – "What do I think will be difficult for me?" | 15 |
| Injection Technique, Glycaemia, and Carbohydrate Counting | Learning injection techniques, understanding glycaemia, and counting carbohydrates during meals | 45 |
| Break | | 15 |
| Diabetes Concepts | Introduction to diabetes | 60 |
| Diabetes Treatments | Exploring different types of diabetes and treatments | 45 |
| Break | | 60 |
| Balanced Nutrition | Nutrition education | 60 |
| Diet of the Child and Young Person with DM1 | Focused nutrition education on diabetes in children and young people | 30 |
| Break | | 15 |
| Type I Diabetes | Treatment of type 1 diabetes and different insulin options | 45 |
| Complications | Addressing acute complications – hypo-, hyper-, and acid-ketosis, and their treatments | 30 |
| WebDia-Mundi | Explanation of the WebDia-Mundi project | 30 |
| **DAY 2** | | |
| Summary of the 1st Day | Discussion on the experience of the previous workshop, focusing on living with DM1 | 15 |
| Therapeutic Education | Introduction to therapeutic patient education | 30 |
| Therapeutic and Pedagogical Patient Interview | Understanding the patient and conducting patient interviews | 45 |
| Patient Interview | Guide-Driven Patient Interview | 30 |
| Break | | 15 |
| Various Guides | Sharing various guides | 45 |
| Physical Activity and Diabetes | Exploring insulin adjustments during physical activity | 30 |
| Understanding the Patient | Motivating patients to manage diabetes and handle conflicts | 30 |
| Break | | 60 |
| Preparation for the Day | Planning the day with patients and families | 45 |
| Preparing a Programme | Group preparation of educational materials for children | 60 |
| Presentation of the Pedagogical Sheets | Discussing age-appropriate educational content for children | 15 |
| Break | | 60 |
| Teaching on Nutrition and Injection Technique | Exploring different individual and group animation techniques | 30 |
| *Post-Test* | | 30 |
| Evaluation of the Two Days | Assessing the achievement of objectives set on the first day | 45 |
| **DAY 3** | | |
| Wellcome | Introduction of the participant and overview of the workshop | 30 |
| Knowing diabetes | Determining key concepts of diabetes | 30 |
| Diabetes management | Dynamic workshops covering injection techniques, nutrition, management of hypoglycemia and hyperglycemia, and sports | 80 |
| Break (practical sessions covering: blood sugar levels, injections, and carbohydrate counting) | | 40 |
| Introduction to WebDia-Mundi | Practical session with WebDia-Mundi | 30 |
| Physical Activity | Practical session on physical activity | 30 |
| Break (practical sessions covering: blood sugar levels, injections, and carbohydrate counting) | | 75 |
| Round Table | Sharing experiences, difficulties, and expressing feelings | 45 |
| Sports | Effects of sports on blood sugar levels | 45 |
| Break (practical sessions covering: blood sugar levels, injections, and carbohydrate counting) | | 30 |
| Management of Hypoglycemia and Hyperglycemia | Practical session of lived experiences | 30 |
| Conclusions | Reflecting on acquired knowledge and emotional experiences | 30 |

**Table S2.** Usability of WebDia Mundi reported by healthcare professionals.

|  | n | % |
| --- | --- | --- |
| Overall score^a^ | 76.0 | (59.1 - 85.6) |
| *Perceived operability of the app* |  |  |
| I could use it by myself^b^ |  |  |
| Yes | 17 | 71 |
| No | 5 | 21 |
| I believe that offers benefits^b^ |  |  |
| Yes | 22 | 92 |
| No | 0 | 0 |
| *Perceived ease of use of the app* |  |  |
| Difficulties using the app^b^ |  |  |
| Yes | 1 | 4 |
| No | 22 | 92 |
| Difficulties with carbohydrate counting^b^ | | |
| Yes | 5 | 21 |
| No | 17 | 71 |
| *Satisfaction with the use of the app* | | |
| Instructions were adequate^b^ |  |  |
| Yes | 23 | 96 |
| No | 0 | 0 |
| Recommended app^b^ |  |  |
| Yes | 12 | 50 |
| No | 11 | 46 |

^a^Median, P25 - P75.

^b^Results may not add due to missing values.

**Table S3.** Differences between the quality of life reported by children and their parents/caregivers at baseline and 3 months later.

|  | Children living with T1DM | Caregivers | *P* |
| --- | --- | --- | --- |
| **Baseline (median, P25 - P75)** | | | |
| Diabetes Symptoms | 59.1 (56.8 - 77.3) | 65.9 (50.0 - 75.0) | .825 |
| Treatment I | 56.3 (50.0 - 81.3) | 50.0 (37.5 - 68.8) | .059 |
| Treatment II | 67.9 (50.0 - 82.1) | 60.7 (46.4 - 78.6) | .353 |
| Worry | 41.7 (25.0 - 50.0) | 41.7 (33.3 - 50.0) | .777 |
| Communication | 66.7 (33.3 - 91.7) | 54.2 (16.7 - 83.3) | .332 |
| *Overall* | 60.5 (43.9 - 69.7) | 56.9 (42.6 - 68.9) | .148 |
| **3 months follow-up (median, P25 - P75)** | | | |
| Diabetes Symptoms | 54.6 (50.0 - 72.7) | 50.0 (50.0 - 59.1) | .229 |
| Treatment I | 50.0 (50.0 - 75.0) | 50.0 (50.0 - 75.0) | .593 |
| Treatment II | 53.6 (50.0 - 78.6) | 50.0 (50.0 - 75.0) | .454 |
| Worry | 50.0 (50.0 - 50.0) | 50.0 (50.0 - 58.3) | .528 |
| Communication | 50.0 (50.0 - 75.0) | 50.0 (50.0 - 75.0) | .748 |
| *Overall* | 61.0 (52.3 - 70.9) | 62.4 (56.4 - 75.8) | .831 |

Wilcoxon matched pairs test was used.

**Table S4.** Percentage of agreement regarding the satisfaction of the workshops and the usability of the WebDia Mundi application.

|  | Healthcare workers | Children living with T1DM | Caregivers |
| --- | --- | --- | --- |
| **Workshop satisfaction (n, %)** | | | |
|  | (n = 24) | (n = 19) | (n = 19) |
| Learned something^a^ | | | |
| Yes | 23 (96) | 14 (74) | 16 (84) |
| No | 0 (0) | 0 (0) | 0 (0) |
| Considered changes after the workshop^a^ | | | |
| Food related | 18 (75) | 11 (58) | 14 (74) |
| Use WebDia Mundi | 2 (8) | 2 (11) | 2 (11) |
| Did not understand | 0 (0) | 1 (5) | 0 (0) |
| Would attend the workshop again^a^ | | | |
| Yes | 20 (83) | 14 (74) | 16(84) |
| No | 0 (0) | 0 (0) | 0 (0) |

^a^Results may not add due to missing values.

**Table S5.** Questions developed by the research team for knowledge perception and workshop satisfaction

| **Target group** | **Question** | **Response format** |
| --- | --- | --- |
| Knowledge perception | | |
| Caregivers | How would you rate your level of knowledge about type 1 diabetes? | Likert scale (very good – very poor) |
|  | How would you rate your level of knowledge about carbohydrate counting? |  |
|  | How would you rate your level of knowledge about insulin management in people with type 1 diabetes? |  |
| Patients (children/adolescents) | How would you rate your level of knowledge about carbohydrate counting? |  |
| Workshop satisfaction | | |
| Health care workers | How did you find the training sessions and workshop? | Likert scale (not interesting – very interesting) |
| Patients and caregivers | How did you find the workshop? |  |
| All participants | What did you like the most about the workshop? | Open-ended |
|  | What did you like the least about the workshop? |  |
|  | Do you plan to make any changes in your daily practice after the workshop? |  |
|  | What did you think about the duration of the workshop? |  |
|  | Any additional comments? |  |
